# Sonoporation of Cells by a Parallel Stable Cavitation Microbubble Array

**DOI:** 10.1002/advs.201900557

**Published:** 2019-06-17

**Authors:** Long Meng, Xiufang Liu, Yuchen Wang, Wenjun Zhang, Wei Zhou, Feiyan Cai, Fei Li, Junru Wu, Lisheng Xu, Lili Niu, Hairong Zheng

**Affiliations:** ^1^ Paul C. Lauterbur Research Center for Biomedical Imaging Institute of Biomedical and Health Engineering Shenzhen Institutes of Advanced Technology Chinese Academy of Sciences 1068 Xueyuan Avenue Shenzhen 518055 China; ^2^ CAS Key Laboratory of Health Informatics Shenzhen Institutes of Advanced Technology Chinese Academy of Sciences 1068 Xueyuan Avenue Shenzhen 518055 China; ^3^ Sino‐Dutch Biomedical and Information Engineering School Northeastern University 195 Innovation road Shenyang 110169 China; ^4^ Faculty of Engineering and Architecture Ghent University Jozef Plateaustraat 22 9000 Ghent Belgium; ^5^ Key Laboratory of E&M Ministry of Education & Zhejiang Province Zhejiang University of Technology 18 Chaowang Road Hangzhou 310014 China; ^6^ Department of Physics University of Vermont Burlington VT 05405 USA

**Keywords:** acoustic radiation force, membrane permeability, sonoporation, stable cavitation, ultrasound bioeffects

## Abstract

Sonoporation is a targeted drug delivery technique that employs cavitation microbubbles to generate transient pores in the cell membrane, allowing foreign substances to enter cells by passing through the pores. Due to the broad size distribution of microbubbles, cavitation events appear to be a random process, making it difficult to achieve controllable and efficient sonoporation. In this work a technique is reported using a microfluidic device that enables in parallel modulation of membrane permeability by an oscillating microbubble array. Multirectangular channels of uniform size are created at the sidewall to generate an array of monodispersed microbubbles, which oscillate with almost the same amplitude and resonant frequency, ensuring homogeneous sonoporation with high efficacy. Stable harmonic and high harmonic signals emitted by individual oscillating microbubbles are detected by a laser Doppler vibrometer, which indicates stable cavitation occurred. Under the influence of the acoustic radiation forces induced by the oscillating microbubble, single cells can be trapped at an oscillating microbubble surface. The sonoporation of single cells is directly influenced by the individual oscillating microbubble. The parallel sonoporation of multiple cells is achieved with an efficiency of 96.6 ± 1.74% at an acoustic pressure as low as 41.7 kPa.

## 1 Introduction

Transient and reparable modulation of membrane permeability that allows for foreign substances to enter cells is of great significance in drug delivery, genome editing, and gene therapy.^1^, ^2^, ^3^ Generally, there are two categories of delivery techniques in applications, viral and nonviral vectors.^4^, ^5^, ^6^ The viral vector method offers high transfection efficiency but may induce undesired side effects such as cytotoxicity and host immunogenicity.^7^, ^8^, ^9^ Nonviral vectors take advantage of the external force field to enhance the membrane permeability and have gained increasing attention due to lower toxicity and immunogenicity than viral vectors.^10^

Sonoporation is a nonviral technique that is capable of generating transient pores in the cell membrane by acoustic cavitation assisted by the presence of microbubbles.^11^, ^12^, ^13^, ^14^ Gas‐filled microbubbles that serve as cavitation nuclei are essential for stable cavitation and sonoporation. Typically, a microbubble's shell is made of lipids, polymers, or proteins. An inert gas such as SF_6_, C_3_F_8_, or C_4_F_10_ is encapsulated in the shell, which stabilizes the inert gas against dissolution and coalescence.^15^, ^16^ The fabrication of microbubbles is based on the mechanical agitation method and the fabricated microbubbles have a wide size distribution, from 1 to 8 µm.^17^, ^18^ When the microbubbles are exposed to a high‐intensity acoustic field, they grow in volume and then collapse violently, known as inertial cavitation (formerly called transient cavitation). During the implosive collapse of the microbubbles, violent physical phenomenon involving jetting, shock wave, and temperature elevation occur, leading to enhancement of the membrane permeability. In noninertial cavitation (also called stable cavitation), microbubbles are forced to oscillate with a relatively small deformation when the pressure amplitude of the acoustic filed is not too high. Although it is initially believed that inertial cavitation is required to generate pores in the cell membrane, increasing evidence indicates that linear and nonlinear oscillation of microbubbles induced by stable cavitation improves the membrane permeability.^19^ Meijering et al. demonstrated that cells exposed to stable cavitation could uptake model drugs with various molecular weights from 4.4 to 500 kDa using 1 MHz pulsed ultrasound with an acoustic pressure of 0.22 MPa.^20^ Compared with inertial cavitation, sonoporation induced by stable cavitation is moderate and controllable and thus has received increasing recent attention.

As the resonant frequency of the microbubbles is highly dependent on the microbubble size, few of the microbubbles oscillate at resonant frequency when the microbubbles are exposed to ultrasound stimulation with a single frequency.^21^, ^22^, ^23^ Oscillating microbubbles of different sizes show a significant difference in amplitude under single ultrasound frequency and thus affect the membrane permeability differently.^24^ Morgan et al. demonstrated significant differences in the oscillation amplitude of cavitation microbubbles with different radii; larger microbubbles (1.3 µm radius) produced a stronger oscillation amplitude than smaller microbubbles (0.7 µm radius) when the transducer excitation was a one‐cycle pulse with an acoustic pressure of 110 kPa and a center frequency of 2.4 MHz.^21^ Fan et al. showed that larger microbubbles exhibited larger responses to the same peak acoustic pressure of 0.17 MPa at 1.25 MHz and tended to enhance membrane permeability.^25^ Consequently, the size uniformity of microbubbles significantly influences the sonoporation efficiency.

The relative distance between the microbubble and cell also plays an important role in the acoustic cavitation‐induced sonoporation.^26^, ^27^, ^28^ Zhou et al. showed that the impact of the microbubble on the membrane permeability decreased dramatically with increasing distance between the cell and the bubble.^29^ Meng et al. found the effective region of the microbubble is extremely small, less than 0.68 the diameter of the microbubble.^30^ Thus, to achieve high sonoporation efficiency, it is necessary to initiate bubble cavitation close to the cells. When the cells are far from the cavitation bubble, the cavitation bubble has no effect on the cell membrane permeability.

With the development of micro‐ and nanofabrication processes, acoustic devices based on microfluidics have been developed to modulate the membrane and investigate the ultrasound bioeffects.^31^, ^32^, ^33^, ^34^, ^35^, ^36^ Due to the flow characteristics of microfluidic devices, more intensive work was focused on the sonoporation of suspended cells. Gac et al. showed that HL60 suspended cells could be sonoporated by a single laser‐induced bubble collapse.^37^ Carugo et al. found that cells could be sonoporated by the acoustic radiation force in a standing acoustic field in the absence of microbubbles and high cell viability could be maintained.^38^ With a combination acoustic field with an electronic field, transient pores could be generated along two axes of the cell membrane, improving the delivery efficiency.^39^ Microfluidic platforms are a powerful tool to enhance membrane permeability, drug delivery, and gene transfection.

**Figure 1:**
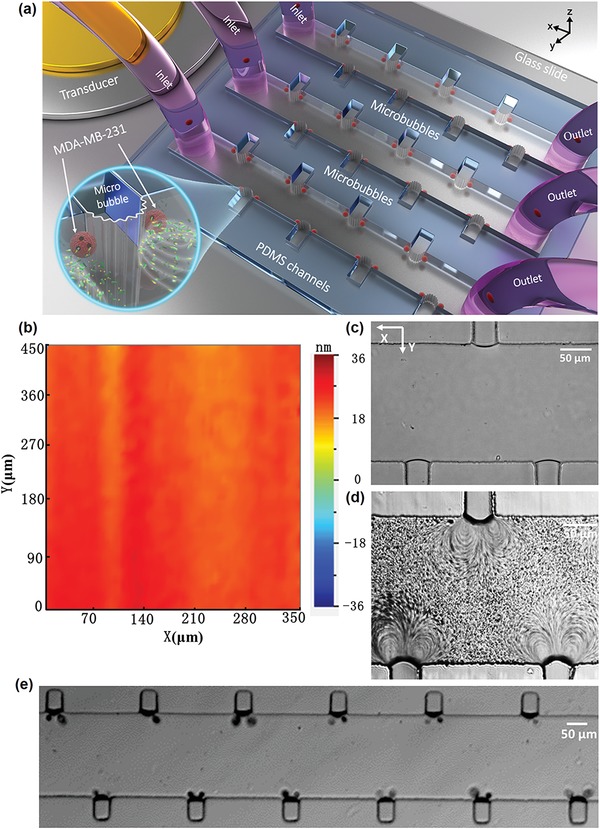
a) Schematic of the experimental setup. The PZT is placed adjacent to the microfluidic channels and excites microbubble oscillation. The microbubbles are generated at microcavities due to the surface tension. Inset: microstreaming is generated by the stable cavitation and the trapped cells are sonoporated by the oscillating microbubbles. b) The vibration amplitude of the glass substrate, measured by LDV, is relatively uniform. c) An optical image of an air microbubble array with the same 40.8 µm diameter. d) An optical image of microstreaming produced by each oscillating microbubble, indicating the independent oscillation of microbubbles. e) Optical image of cells trapped by a microbubble array.

Although sonoporation technology has been substantially developed, there are challenges that should be overcome to promote practical applications: i) the nonuniformity of the microbubble size distribution, ii) controlling the relative distance between the cells and microbubbles, and iii) maintaining the microbubble at stable cavitation. In this study, we developed a microfluidic device with multirectangular structures that enables self‐repairable sonoporation at the single‐cell level in a parallel manner, as shown in **Figure** **1**a. When the piezoelectric transducer (PZT) is excited, the vibration amplitude of the glass substrate within the microchannel is relatively uniform, as depicted in Figure 1b, measured by a laser Doppler vibrometer (LDV; UHF‐120, Polytec GmBH, Waldbronn, Germany). Due to the surface tension, an array of air microbubbles with the same diameter can be generated when the fluid flows through the microchannel (Figure 1c and Movie S1, Supporting Information). Individual microbubbles oscillate and generate microstreaming independently, as shown in Figure 1d. The passive cavitation detector (PCD) based on an LDV system indicated that stable cavitation occurs. Together with the secondary acoustic radiation force and drag force induced by the microbubble oscillation, single cells can be trapped at the microbubble surface. The shear stress induced by the individual oscillating microbubbles deforms the membrane and modulates the membrane permeability. By designing parallel microchannels with multiple microbubbles, high‐efficiency sonoporation was achieved.

## 2 Results

### 2.1 Microstreaming Induced by Oscillating Microbubbles

An air microbubble can be generated at the interface between the polydimethylsiloxane (PDMS) and the fluid and trapped within the rectangular microcavity (Figure 1c). The resonance frequency (*f*) of a trapped microbubble in stationary fluid is estimated by the small‐amplitude behavior of the Rayleigh–Plesset equation^40^, ^41^

(1) $$f^{2}=\frac{1}{4\rho_{f}\pi^{2}R_{b}{}^{2}}\left\{3k\left(p+\frac{2\sigma }{R_{b}}\right)-\frac{2\sigma }{R_{b}}\right\}$$

where ρ_f_ is the density of the phosphate buffer saline (PBS) solution, σ is the surface tension of the PBS solution, *k* is the polytropic exponent for a bubble containing air, *p* is the static pressure, and *R*_b_ is the radius of the bubble. The frequency is calculated as 167.5 kHz (Note 1, Supporting Information) using Equation (1). Experimentally, a series of PZT transducers with a resonant frequency of 100–200 kHz with an increment of 10 kHz were utilized to excite microbubble oscillation. Prominent microstreaming induced by microbubble oscillation was observed at 107 kHz and thus the exciting frequency of 107 kHz was chosen for all the experiments. The differences between the experimental and theoretical frequencies are mainly attributed to the hemisphere‐shaped air bubbles in the experiment, while Equation (1) is based on a spherical bubble.

**Figure 2:**
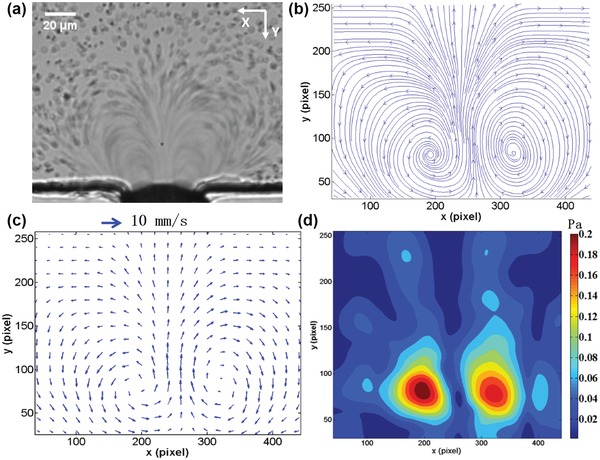
a) Optical image of microstreaming induced by microbubble oscillations. When the PZT is excited, the 2 µm polystyrene particles around the microbubble are driven to follow the microstreaming. The trajectory of the particles is demonstrated in Movie S2 in the Supporting Information. b,c) The streamline and velocity vector of the flow field traced by PIV analysis. The streamlines and velocity vector images indicate that two symmetrical vortexes are generated in the fluid field induced by the oscillating microbubble. d) Illustration of the distribution of shear stress induced by microstreaming. The maximum shear stress occurs in the central area of the vortices.

To visualize the microstreaming induced by the microbubble oscillation, a solution of polystyrene particles with a diameter of 2 µm was injected into the microchannel.^42^ The 2 µm particle tracer was small enough to follow the flow streaming with reasonable precision, much smaller than the wavelength of the excitation frequency of approximately 0.63 mm, and can be used to characterize the fluid field. When the trapped microbubble is excited with an acoustic pressure of 41.7 kPa at 107 kHz, the acoustic energy is coupled with the microbubble and the microbubble starts to oscillate immediately. **Figure** **2**a shows how particles within the microstream follow two symmetric near‐ellipsoidal trajectories, and the velocity of the particles changes dramatically at various positions (Movie S2, Supporting Information). To quantitatively analyze the fluid field resulting from the microstreaming, particle image velocimetry (PIV) method was utilized to measure the flow pattern.^43^, ^44^ Figure 2b,c shows the streamlines and velocity vectors of the fluid field, respectively. The microstreaming field contained two closed‐loop rotational flows in a plane perpendicular to the substrate and the maximum streaming velocity was 7.5 mm s^−1^. The streaming pattern induced by the single microbubble oscillation qualitatively agreed with those described by Marmottant and Hilgenfeldt.^45^ The effective distance of the microstreaming in the *X*‐direction was approximately 130 µm, 3.25‐fold the microbubble diameter, which agrees with the study by Marin et al.^46^ However, the effective region was smaller than that of microstreaming induced by a commercial ultrasound contrast agent (SonoVue), approximately 2 mm, demonstrated by Pereno et al.^47^ The maximum velocity of the microstreaming generated by the oscillation of air bubbles and SonoVue was on the same order, 1 mm s^−1^. The shear stress arising from the microstreaming can be estimated as follows^48^

(2) $$\tau \;=\;\mu \sqrt{{\left(\frac{\partial \gamma \;}{\partial y}\right)}^{2}+{\left(\frac{\partial v}{\partial x}\right)}^{2}}$$

where μ is the dynamic viscosity and γ and *v* are the flow velocity in *x*‐ and *y*‐directions, respectively. As depicted in Figure 1d, the maximum shear force is approximately 0.2 Pa at the central area of the vortices and decreases as the contour diverges. Since the oscillation of the microbubble is relatively stable, inertial cavitation is avoided and the repeatability of the results is ensured.

### 2.2 Cells Trapped by Oscillating Microbubbles

**Figure 3:**
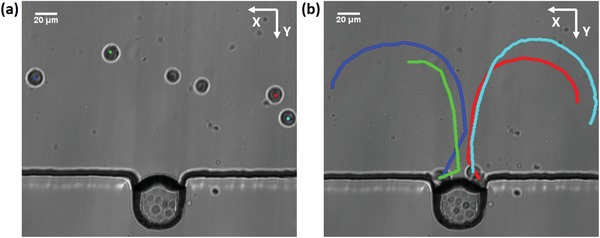
Cells are trapped by the microbubble oscillations. a) Prior to the application of ultrasound, the cells are distributed randomly in the channel. The solution of harvested MDA‐MB‐231 cells is injected to the microchannel. b) Moving trajectory of the cells within the microstreaming. With the presence of ultrasound, cells are attracted to the proximity of the bubble membrane and trapped there.

A solution of digested breast cancer MDA‐MB‐231 cells (10^6^ cells mL^−1^) was introduced into the microfluidic channel by a syringe pump (neMESYS, Cetoni, Germany) and the cells were distributed randomly, as shown in **Figure** **3**a. The syringe pump was turned off and the sonoporation experiments were carried out in a still flow field. With the presence of ultrasound, the microbubbles began to oscillate and the cells in a quiescent flow field were trapped at microbubble surface within a second (Movie S3, Supporting Information). Figure 3b shows the movement trajectories of cells within the microchannel, which agrees with the streamlines of the microstreaming measured by the PIV. When the acoustic pressure was 41.7 kPa, the effective range of the microstreaming in the *Y*‐direction was approximately 200 µm. With increasing input power, the effective range of microstreaming to cells increases correspondingly.

The trapping mechanism can be attributed to two primary forces applied to cells induced by the oscillating bubble. The cells experience both microstreaming‐induced drag force and the secondary acoustic radiation force generated by the oscillating bubble. The drag force induced by microstreaming directs the cell movement along the streamline in the fluid, as shown in Figure 3b. The drag force on a cell can be described as^49^

(3) $$F_{d}=\;6\pi \mu R_{c}U$$

where *U* and *µ* are the relative streaming velocity and the dynamic viscosity of the PBS solution, respectively. *R*_c_ is the radius of the cell. The maximum velocity in the microstreaming is approximately 7 mm s^−1^. The maximum drag force was calculated as 1.3 nN (Note 2, Supporting Information). The secondary radiation force, normally called the Bjerknes force, originates from the pulsating air bubble and can be estimated by^50^, ^51^

(4) $$F_{r}=\;4\pi \frac{\rho_{f}\;-\;\rho_{p}}{\rho_{f}\;+2\rho_{p}}\frac{R_{b}{}^{4}R_{c}^{3}}{d^{5}}\omega^{2}\varepsilon^{2}$$

where *R*_b_ is the radius of a microbubble, *d* is the distance between the centers of the microbubble and the cell, ω is the angular driving frequency, ε is the oscillating amplitude of the microbubble, and ρ_f_ and ρ_p_ are the densities of the PBS solution and the particle, respectively. The maximum secondary acoustic radiation force is 11 nN when the cells are contracted from the microbubble surface (Note 3, Supporting Information). As shown in Equation (4), the direction of the radiation force can be attractive or repulsive, and is determined by the relative density between the surrounding medium and the particles. Higher density particles (ρ_f_ > ρ_p_) are attracted towards the oscillating bubble whereas particles with a density lower than that of the medium are repelled (ρ_f_ < ρ_p_). Consistent with the theoretical results, MDA‐MB‐231 cells in the experiments were attracted toward the oscillating bubble due to their higher density.^52^, ^53^ According to Equation (3), the magnitude of the secondary acoustic radiation force is highly dependent on the relative distance between the particles and the bubble. When the cells are far from the microbubble, the drag force plays a dominant role compared to the secondary acoustic radiation force, resulting in movement of the cells in line with the streamline. As the cells approach the microbubble, the radiation force increases dramatically $\left(F_{r}\propto \frac{1}{d^{5}}\right)$ and the secondary acoustic radiation force eventually becomes predominant. The initiated cells are accelerated toward the microbubble and trapped at the surface of the microbubble membrane.

### 2.3 Modulation of Cellular Permeability

**Figure 4:**
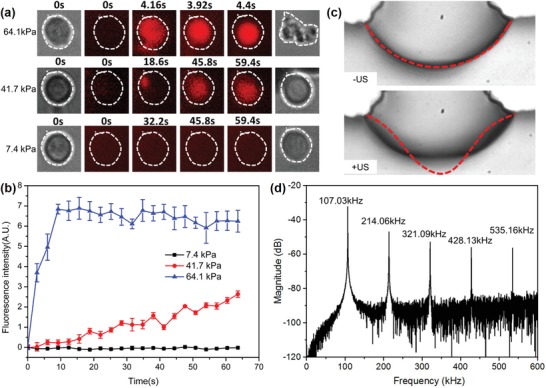
a) Sonoporation process at various input acoustic amplitudes. b) Quantitative analysis of the PI fluorescence intensity as a function of time (*n* = 5). c) Membrane deformation of the oscillating microbubble when the acoustic pressure is 41.7 kPa. d) Scattering signal induced by microbubble oscillation. Higher harmonics are detected by PCD, illustrating that stable (noninertial) cavitation occurs.

Suspended MDA‐MB‐231 cells in a solution with propidium iodide (PI, Sigma‐Aldrich) were injected into the microchannel. The solution of PI was utilized to determine sonoporation events as it can only pass through the damaged cell membrane, generating red fluorescence. Bright field imaging and fluorescence imaging were performed in place to visualize the bubble oscillation, cell movement, and changes in membrane permeability in real time. The cell outline was labeled with a white dotted line and the PI pattern was measured as the average fluorescence intensity within the dotted line region. Prior to the application of ultrasound, no fluorescence was observed initially (*t* = 0), illustrating that the cellular membrane was intact. **Figure** **4**a shows the process of cellular sonoporation at various acoustic pressures (Movie S4, Supporting Information). When the acoustic pressure was 64.1 kPa, the microbubble membrane began to oscillate violently. Cells were immediately trapped at the bubble surface. Red fluorescence due to PI uptake was observed (Figure 4a). Figure 4b shows the fluorescence intensity curve of the PI uptake within the cytoplasm over time. The results show that the peak fluorescence signal can be achieved within 10 s after the microbubble starts to oscillate. The bright field image shows that the intact cell was broken into multiple cellular fragments. When the acoustic pressure was 41.7 kPa, the oscillation amplitude of the bubble membrane was relatively smaller, leading to a gradual increase in PI intensity. By increasing the acoustic pressure to 53.6 kPa, similar results were achieved with a smaller treatment time. The morphology of the cell does not show significant changes after bubble oscillations stop. For the sonoporation, the optimized acoustic pressure was 41.7–53.6 kPa. Once the cells were sonoporated, the input voltage applied to the PZT was shut off. The cells trapped at the microbubble surface were released from the microbubble surface (Figure S1, Supporting Information) and approximately 98.46 ± 1.54% cells were released. During the process of microbubble oscillation, periodical local surface deformation with an amplitude of 3 µm was recorded by a high‐speed charge‐coupled device (CCD) with a frame of 100 kHz, as shown in Figure 4c. When the cell is trapped, the shear stress in the vicinity of a pulsating bubble can be estimated using the following equation^54^, ^55^

(5) $$S\;=\;2\pi^{3/2}\varepsilon^{2}{\left(\rho_{f}f^{3}\mu \right)}^{1/2}/R_{b}$$

Using this equation, the shear stress on the cell is calculated as 177 Pa (Note 4, Supporting Information). To verify whether acoustic cavitation occurs, a passive cavitation detection (PCD) system was installed. Figure 4d shows the fast Fourier spectrum of the signals detected by the PCD, with multiple higher harmonic components in addition to the fundamental resonant frequency signal. The broadband noise and subharmonic peaks associated with inertial cavitation were not observed, indicating that the cavitation is a stable cavitation. When the acoustic pressure is decreased to 7.4 kPa, no PI fluorescence signal was detected, indicating that the permeability of the membrane was not compromised.

### 2.4 Parallel Sonoporation of Cells

**Figure 5:**
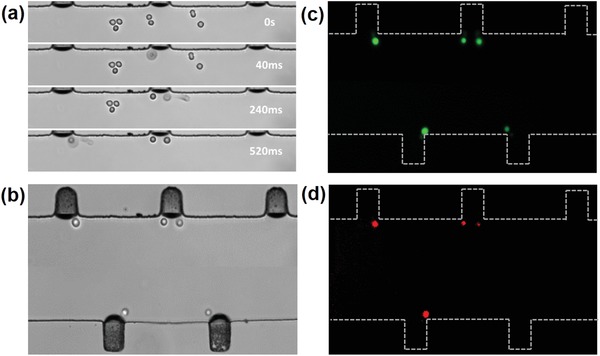
a) With the presence of ultrasound, all cells in the microchannel are attracted toward the oscillating microbubbles in 520 ms. b) Bright‐field image of a single cell trapped at an oscillating microbubble surface. c,d) Fluorescence images of the membrane permeability of 88.89 ± 1.53% cells are enhanced by their corresponding oscillating microbubble and almost all the cells remain viable (*n* = 5).

Multirectangular cavities in the main channel of a fluidic device trap microbubbles, which are excited by ultrasonic waves to generate sonoporation of cells. As the rectangular cavity has a uniform size, the microbubbles generated by the surface tension in each microcavity have the same diameter of 40.8 µm. The size of the microbubbles is nearly a monodispersive distribution, with a standard deviation of 4.6%. As the effective region of a single bubble in the *X*‐direction is approximately 130 µm (acoustic pressure 41.7 kPa), the edge‐to‐edge spacing between two nearest individual rectangular cavities was designed to be 240 µm, which is far enough. The interaction of the nearest neighbor bubbles can be ignored, ensuring that microstreaming induced by each oscillating microbubble is independent. To improve the capture efficiency, the rectangular cavities are arranged in an alternating pattern within the main channel (Figure 1e). With the presence of ultrasound, a single cell in the microchannel is attracted toward its corresponding microbubble immediately within 0.6 s, as shown in **Figure** **5**a. Almost all cells suspended in the microchannel are trapped (Figure 5b) and the cell trapping rate reached 95.50 ± 2.78% (*n* = 5).

A fatality test of the sonoporated cells was performed using a calcein‐AM/PI double staining assay.^56^ Due to the uniformity of the bubble size, the maximum streaming velocity and shear stress induced by individual oscillating bubbles at various locations are approximately the same (Figure S2, Supporting Information). Thus, the trapped cells experience the same order of magnitude of shear stress caused by individual oscillating bubbles, leading to the homogeneous sonoporation of each cell. Figure 5c,d shows that 88.89 ± 1.53% of the cells' membrane permeability was enhanced, as almost all the cells emitted green fluorescence. The figure also shows that the cell viability was not altered during the sonoporation process.

**Figure 6:**
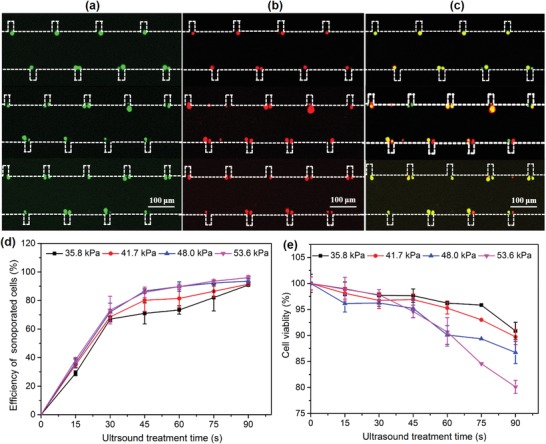
a) Single cells trapped at the oscillating microbubble array emit green fluoresce, confirming the cell viability. b) Trapped single cells emit red fluoresce under the action of oscillating microbubble array. c) Merged fluorescence image showing that high sonoporation efficiency can be achieved by a parallel stable cavitation microbubble array. d,e) Sonoporation efficiency and cell viability at various acoustic pressures as a function of ultrasound treatment time.

To verify the high efficacy of sonoporation using the parallel sonoporation technique, a multichannel with the same structure was designed, as shown in Figure 1a. When the PZT transducer is connected to the output of the power amplifier, the ultrasonic wave propagates along the glass substrate, leading to the oscillation of the substrate. Figure 1b shows the vibration amplitude of the glass substrate within the microchannel. The vibration amplitude at various positions in the microchannel is relatively uniform and thus the corresponding acoustic pressure in the microchannel is almost uniform, with a 5% variation. Under the action of individual bubble oscillations, all cells suspended in each channel are trapped and emitted green fluorescence (**Figure** **6**a). The enhancement of the membrane permeability of individual cells is shown in Figure 6b. Figure 6c is a merged fluorescence image of calcein‐AM/PI showing that the cells at various microchannels are sonoporated efficiently. Figure 6d shows the sonoporation efficiency at various acoustic pressures with the sonication time. The results show that the sonoporation efficiency increases with increasing acoustic pressure and ultrasound treatment time. High sonoporation efficiency of 96.6 ± 1.74% is achieved when the acoustic pressure is 53.6 kPa with a treatment time of 90 s. However, a decline in cell viability was observed with increasing acoustic pressure and ultrasound treatment time. The cell viability decreased from 97.9 ± 1.26% to 80.11 ± 1.19% when the treatment time was increased from 30 to 90 s (acoustic pressure 53.6 kPa). To further investigate the long‐term cell viability, the sonoporated cells were collected and cultured in 96‐well plates for 0, 24, and 48 h. Optical imaging and a cell‐counting Kit‐8 (CCK‐8, DOJINDO, Japan) were used to examine the cell viability and proliferation (Figures S3 and S4, Supporting Information). Compared with the control group, no significant change in the morphology and viability was detected after 24 and 48 h when the acoustic pressure was 7.4 and 41.7 kPa. With an acoustic pressure of 64.1 kPa, the cell viability sharply decreased to 3.47%.

## 3 Discussion

Applications of stable sonoporation assisted by microbubbles hold great potential for efficient, localized safe drug/gene delivery.^57^ To achieve highly localized drug concentrations, it is crucial to enhance the cellular membrane permeability instantaneously, increasing drug influx through the membrane during the drug delivery process. Previous studies have indicated that, compared with inertial cavitation, the process of stable cavitation‐induced sonoporation is more controllable and the cell mortality is low.^14^ However, due to the wide size distribution of the microbubbles, the resonant frequencies of the microbubbles vary widely. The oscillation amplitude of microbubbles varies significantly with different microbubble size, which directly influences the sonoporation efficiency. In this paper, we developed a multirectangular structure that can generate microbubbles of almost the same size using surface tension (Figure 1c). The microbubbles appear highly monodispersed, with a standard deviation of 4.6%. The monodispersed microbubbles undergo oscillations with the almost same amplitude with ultrasonic excitation at 107 kHz. Moreover, no broadband noise was observed by the PCD (Figure 4d), illustrating that the microbubbles achieve stable cavitation situations.

Another factor that influences sonoporation is the relative distance between the oscillating microbubble and cells. Ward et al. demonstrated that the percentage of sonoporated cells is proportional to the cubic power of the microbubble–cell spacing.^58^ For stable cavitation‐induced sonoporation, previous studies indicated that it is essential for cells to contact the oscillating microbubbles directly. By combining the secondary acoustic radiation force and the drag force induced by microstreaming, a single cell can be trapped at the microbubble surface at a trapping rate of up to 95.50 ± 2.78% (Figure 5b).^59^, ^60^ Additionally, the air microbubble generated by the air/liquid interface without a shell used in this study can oscillate freely, making the sonoporation more efficient.

The parallel oscillation of individual microbubbles improves the homogeneousness of sonoporation and makes it possible to perforate a single cell with high efficacy. Figure S5 in the Supporting Information shows the sonoporated and collected cells over time when the acoustic pressure was 41.7 kPa. Within 30 s, there were 429.6 ± 3.6 and 696 ± 4.8 sonoporated and collected cells, respectively. Typically, traditional sonoporation of suspended cells is carried out in a single channel and the number of sonoporated cells is less than 100.^54^ The parallel structure in this study enables treatment of multiple cells at once. After 150 s, more than 1863.6 cells were sonoporated. The efficiency of the parallel sonoporation platform can be further improved by designing more microchannels on the glass substrate.

The developed parallel sonoporation method also facilitates investigation of the mechanism underlying the membrane perforation by sonication with oscillating microbubbles. When the microbubble is excited to oscillate at a resonant frequency with a small amplitude, microstreaming can be generated around the oscillating bubble. Marmottant and Hilgenfeldt demonstrated that the shear stress induced by microstreaming is the primary reason for the enhanced cell permeability.^45^ Moreover, Wu et al. demonstrated that the threshold of the shear stress–induced sonoporation was 12 Pa and the exposure time must be longer than 7 min.^55^ When the cells are far from the oscillating microbubble, the shear stress on a cell induced by the microstreaming is approximately 0.2 Pa, which is much lower than the threshold of 12 Pa. When the cells are trapped at the oscillating microbubble surface, the shear stress increases dramatically, to approximately 177 Pa. The shear stress in the vicinity of the oscillating microbubble is large enough to generate pores in the membrane within 30 s due to the larger velocity gradient. Decreasing the acoustic pressure to 7.4 kPa, the amplitude of the bubble oscillation was 7 nm by LDV. In this case, the shear stress applied to the cells is extremely small. Single cells can only be trapped and rotated at the microbubble surface (Movie S5, Supporting Information). No apparent red fluorescence was observed during the whole process, indicating that the membrane is intact even after a longer exposure time (10 min). Therefore, the sonoporation events may be mainly attributed to the shear stress in the vicinity of a pulsating bubble induced by the stable cavitation (Movie S6, Supporting information).

## 4 Conclusion

Ultrasound assisted by cavitating microbubbles enables the generation of transient pores in the membrane and has the potential for drug and gene delivery. The cavitation events appear to be a random process, resulting in a low consistent sonoporation outcome. A new strategy should be developed to improve the homogeneousness, efficiency, stability, and safety of the sonoporation. In this paper, we demonstrated a microfluidic device that is capable of exciting microbubble array oscillation to realize parallel sonoporation at the single‐cell level. In the microbubble array with bubbles of the same size, the microbubbles can undergo stable cavitation by single frequency excitation (107 kHz) and the oscillating amplitude of the individual microbubbles is almost same, ensuring the homogeneousness of the sonoporation. Moreover, the secondary radiation force enables trapping single cells at the microbubble surface. Since the cells contact the microbubbles directly, the shear stress generated by the oscillating microbubble can disturb the membrane effectively and result in a high sonoporation rate of 96.6 ± 1.74%. This parallel device can be an efficient and versatile tool for investigation of the mechanism of sonoporation at the single‐cell level and for potential applications in gene transfection.

## 5 Experimental Section

*Sonoporation Chip*: A PDMS microfluidic chip was designed and fabricated by the standard replica molding technique. The sonoporation device included two parts: a PDMS channel and a glass substrate (Figure S6, Supporting Information). Figure S6a–d in the Supporting Information shows the fabrication process of the PDMS microchannel. Prior to spin coating the photoresist, residual impurities on the surface of the silicon wafer were removed by pickling, alcohol, and water washing. Then, the negative photoresist (SU‐8 50, Microlithography Chemical Corp., Newton, MA) was spin‐coated onto silicon wafers at 500 rpm for 1 min. To volatilize the organic solvent and enhance the adhesion between the photoresist and the silicon wafer, the silicon wafer was placed on a horizontal heating plate at 60 °C for 3 min and 90 °C for 6 min. Subsequently, the photoresist was exposed to a UV light source at 600 cJ cm^−2^ for 30 s and developed in a photoresist developer. Glue A:B (10:1) mixture of PDMS prepolymer and curing agent (Sylgard 184, Dow Corning, Midland, MI) was cast onto the silicon template, degassed under vacuum, and baked at 80 °C for 30 min. The cured PDMS was peeled from the silicon template and the inlet and outlet of the microchannel were created using a puncher (Harris Uni‐Core, Jed Pella, Inc.). Finally, the microchannel was bonded to the glass substrate by plasma treatment. The height of the channel was approximately 50 µm, measured by a step profiler (XP1, MTS, USA), and a rectangular hole was located at the sidewall with a width of 40.8 µm. Both the PDMS channel and glass slide were subjected to oxygen‐plasma treatment and the PDMS channel was then bonded to the glass substrate permanently.

When aqueous solution was injected into the microchannel, air bubbles could be generated and trapped at the rectangular hole due to the discontinuity across the interface between the PDMS and the fluid (Figure 1c, Movie S1, Supporting Information). According to bubble dynamics theory, the resonance frequency of the trapped bubble with a diameter of 40.8 µm was approximately 167.5 kHz. Experimentally, a series of PZT transducers with a resonant frequency of 100–200 kHz were chosen to excite microbubble oscillation with an increment of 10 kHz. Prominent microstreaming induced by microbubble oscillation could be observed at 107 kHz and thus the exciting frequency of 107 kHz was utilized in all experiments. This transducer was fabricated using PZT‐4 ceramic with a diameter of 26 mm and was operated in a thickness vibration mode. The deviations in the resonant frequency between the theoretical and practical results might be due to the semispherical shape of the air bubble trapped in the channel. The ultrasound transducer was adhered to the glass slide with an ultrasonic coupling agent (Guang gong, Guang dong, China), ensuring that the acoustic energy could be efficiently coupled to the substrate. In each experiment, 3 mL ultrasonic coupling agent was applied to the PZT surface, with an average thickness of 3 mm. A continuous sine wave generated by a function generator (AFG 3102, Tektronix, USA), amplified by a power amplifier (2100l, Electronics Innovation, USA) was applied to the transducer to excite the air bubble oscillation.

*Acoustic Parameter Characterization*: The acoustic parameter was commonly measured by a calibrated hydrophone. However, it was difficult to measure the acoustic pressure in an enclosed microsized channel. To address this issue, a LDV (UHF‐120, Polytec, Germany) with a tiny light spot (2.5 µm) was utilized to measure the acoustic pressure within the microchannel in a noncontact manner.^61^ The LDV was positioned perpendicular to the propagation direction of the acoustic waves (Figure S7, Supporting Information) and the LDV could measure the vibration amplitude of the glass substrate based on the Doppler effect. The fluid particle velocity amplitude was assumed to be the same as the particle velocity amplitude of the glass substrate.^62^ The acoustic pressure around an oscillating microbubble could be derived from the following equation

(6) $$P\;=\;2\pi f\rho_{f}c\varepsilon$$

where *f* is the resonance frequency of the PZT, ρ_f_ is the density of the PBS solution, *c* is the sound speed in water, and ε is the vibration displacement of the substrate. The fluid particle velocity amplitude was assumed to be the same as the particle velocity amplitude of the glass substrate. The relationship among the input voltage applied to the PZT, vibration amplitude of the glass substrate, and acoustic pressure is listed in Table S1 in the Supporting Information and the peak‐negative acoustic pressure in the experiments was 7.4–64.1 kPa.

LDV enabled measurement of the vibration amplitude of the glass substrate and the displacement of the oscillating microbubble. Stable cavitation could thus be detected by the LDV‐based PCD method. From the Fourier transform of the displacement of the waveform, the displacement component of the microbubble oscillation at various frequencies could be acquired. If harmonic component signals emitted by the oscillating microbubble were observed, stable cavitation occurred.

*Cell and Particle Preparation*: MDA‐MB‐231 is a triple‐negative breast cancer cell that is widely used in sonoporation experiments.^63^, ^64^, ^65^ The MDA‐MB‐231 was cultured in Dulbecco's modified Eagle medium (Gibco, Life Technologies, Carlsbad, CA, USA) with 10% fetal bovine serum (BI, USA) and 1% v/v penicillin/streptomycin (HyClone, USA) and grown in a 25 cm^2^ cell culture flask (Corning, USA) at 37 °C in a humidified environment with 5% CO_2_. The cells were harvested with 0.25% trypsin (Try, Gibco, USA). The cell concentration was approximately 10^6^ cells mL^−1^ in the experiments. The cells were suspended in PBS solution and injected into the microchannel using a syringe pump (neMESYS, Cetoni, Germany) at 0.3 µL min^−1^. Once the cells were injected into the channel, the syringe pump was shut off. The sonoporation experiment was carried out in a quiescent flow field. The 2 µm polystyrene particles (Sigma‐Aldrich, America) were diluted in 0.1% Tween‐80 deionized water solution to prevent adhering of samples to the substrate.

*Cell Sonoporation and Stain*: PI (P4864, Sigma Aldrich, USA) was added to the medium to detect the sonoporation events. The uptake of PI was utilized to investigate the membrane permeability of the targeted cell. Once PI crossed the cell membrane and bound to DNA and RNA, red fluorescence is emitted. Calcein‐AM (C1359MSDS, Sigma‐Aldrich, USA) was also added to the medium to verify the cellular viability, as it only stains living cells to generate green fluorescence. Per the instructions, the concentration of PI and calcein‐AM in the experiments was 6 and 8 µM, respectively. Prior to the sonoporation experiments, the MDA‐MB‐231 cell suspension was incubated with PI and calcein‐AM for 10 min. The sonoporation efficiency was assessed by the fluorescence imaging method and was calculated using the following formula: Sonoporation efficiency (%) = Count cell‐PI/Count cell‐calcein‐AM × 100% (Count cell‐PI, red cells; Count cell‐calcein‐AM, green cells). Each experiment was repeated five times and the fluorescence images were processed with ImageJ software (8.0, National Institutes of Health).

*Experimental Setup and Data Analysis*: The sonoporation events were captured by a Cool snap CCD digital camera (CoolSNAP HQ2, Photometrics, USA) through an inverted fluorescence microscope (DMI3000B, Leica, Germany) and a laser scanning confocal microscope (TCS SP5, Leica, Germany). A PIV technique was used to quantitatively characterize the microstreaming induced by oscillation of the air bubbles. Video of the acoustic streaming was captured by a high‐speed CCD camera (MC1310, Mikrotron, Germany) at 500 frames s^−1^ (exposure time: 2 ms; gain: 1; objective: 20×). An ultrafast CCD camera (FASTCAM SA‐X2, model 1000K‐M2) was employed to record the deformation of the microbubble at 100 kHz.

*Statistical Analysis*: All data were expressed as the mean ± SEM. An independent sample *t*‐test and analysis of variance were implemented and used for comparison between multiple groups using the statistics software SPSS ver.12.0. The level of statistical significance was set at *p* < 0.05.

## Conflict of Interest

The authors declare no conflict of interest.

## Supporting information

Supplementary

Supplementary

Supplementary

Supplementary

Supplementary

Supplementary

Supplementary
