# Supplementary material for: Sonoporation of Cells by a Parallel Stable Cavitation Microbubble Array
Source: Adv Sci (Weinh). 2019 Jun 17;6(17):1900557. doi: 10.1002/advs.201900557 (PMC6724477; doi:10.1002/advs.201900557)
Supplement: Supplementary file 1 — Supplementary [file ADVS-6-1900557-s002.pdf]

Copyright WILEY-VCH Verlag GmbH & Co. KGaA, 69469 Weinheim, Germany, 2019.

ADVANCED SCIENCE
Open Access

Supporting Information

for *Adv. Sci.,* DOI: 10.1002/advs.201900557

Sonoporation of Cells by a Parallel Stable Cavitation Microbubble Array

Long Meng, Xiufang Liu, Yuchen Wang, Wenjun Zhang, Wei Zhou, Feiyan Cai, Fei Li, Junru Wu, Lisheng Xu, Lili Niu, and Hairong Zheng**

Note 1: The resonance frequency of small amplitude for a microbubble

The resonance frequency (***f***) of a trapped microbubble with small amplitude in stationary fluid is estimated from the following equation derived from the Rayleigh–Plesset equation:

$$\boldsymbol{f}^2 = \frac{1}{4\rho_f \pi^2 {R_b}^2}\left\{3k\left(p + \frac{2\sigma}{R_b}\right) - \frac{2\sigma}{R_b}\right\} \qquad (1)$$

where ρ_f is the density of the phosphate buffer saline (PBS) solution, σ is the surface tension of water, k is the polytropic exponent for a bubble containing air, p is the fluidic pressure, and R_b is the radius of the bubble. The frequency is calculated to be 167.5 kHz by using **Equation (1)** ($\rho_f = 1148\, kg\, m^{-3}, \sigma = 0.07179\, N\, m^{-1}, k = 1.4, p = 100\, kPa, R_b = 20\, \mu m$).

Note 2: Theoretical value of drag force on cells

The maximum shear stress is measured to be approximately 0.2 Pa using particle image velocimetry (PIV) method corresponding to 60 pN. The **drag force** could expressed as:

$$\boldsymbol{F_d} = 6\pi\mu R_c U \qquad (2)$$

where U and μ are the relative streaming velocity and the dynamic viscosity of the fluid respectively. R_c is the radius of the cell. The maximum velocity in the microstreaming was approximately 7 mm/s. The maximum drag force was calculated to 1.3 nN by using **Equation (2)** ($U = 7mm\, s^{-1}$, $\mu = 1010\, pa.s, R_c = 10\, \mu m$).

Note 3: Theoretical value of secondary acoustic radiation force on cells

The secondary acoustic radiation force $\boldsymbol{F_r}$ is estimated by:

$$\boldsymbol{F_r} = 4\pi \frac{\rho_f - \rho_p}{\rho_f + 2\rho_p} \frac{{R_b}^4 R_c^3}{d^5} \omega^2 \varepsilon^2 \quad (3)$$

where R_b is the radius of a microbubble, d is the distance between the centers of the nearby microbubble and cells, ω is the angular driving frequency, ε is the oscillating amplitude of the microbubble, ρ_f and ρ_p are the density of PBS solution and particle, respectively. In the experiments, R_b is measured to be 3.02 μm by LDV. The maximum secondary acoustic radiation force, $\boldsymbol{F_r}$ is 11 nN when the cells is contracted near the microbubble surface ($R_b = 20\ \mu m, R_c = 10\ \mu m\ , d = 30\ \mu m\ , \omega = 2\pi f \sim 671.63\ \mathrm{rad\ s^{-1}}, \rho_f\ =\ 1148\ kg\ m^{-3},\ \varepsilon = 3.02\ \ \mu m$).

Note 4 Theoretical value of shear stress on the trapped cell

For the sonoporation procedure, once the cells is trapped, the **shear stress** in the vicinity of a pulsating bubble could be given:

$$\boldsymbol{S} = 2\pi^{3/2} \varepsilon^2 (\rho_f f^3 \mu)^{1/2} / R_b \quad (4)$$

According to this **Equation (4)**, the shear stress on the cell is about 177 Pa ($\varepsilon = 3.02\ \mu m, \rho_f = 1148\ kg\ m^{-3}, f = 107\ kHz, \mu = 1010\ Pa.s,\ \ R_b = 20\ \mu m$).

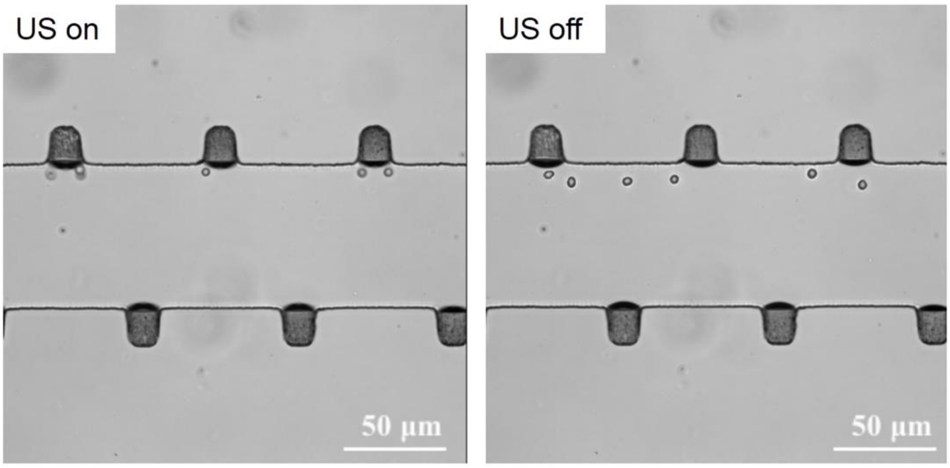

Figure S1 Cells are trapped on microbubble surface and released from microbubble surface. (a) When input voltage is applied to PZT, cells in fluid are trapped to the microbubble surface immediately. (b) Once the input voltage applied to PZT is shut off, the trapped cells are released from the microbubble surface.

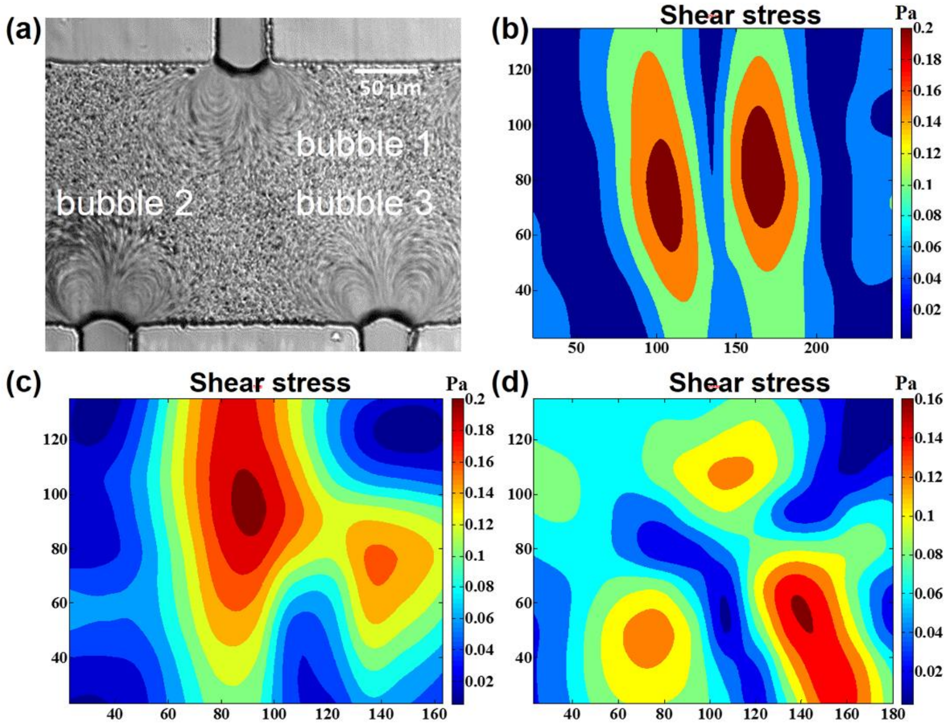

Figure S2 Photos show characteristics of the streaming fields of multiple bubbles located at different distances from the transducer. (a) Three streaming fileds are generated by oscillating microbubbles located at different locations (bubble1, bubble 2 and bubble 3). (b-d) The distribution of shear stress induced by bubble 1, bubble 2 and bubble 3 respectively. The maximum shear stress resulted from the bubble 1, bubble 2 and bubble 3, is 0.2 Pa, 0.2 Pa and 0.16 Pa, respectively. It is demonstrated that no significant change in shear stress is found when the oscillating microbubbles are located at various positions. Moreover, the maximum streaming velocity also has the same order of magnitude; the maximum streaming velocity generated by the bubble 1, bubble 2 and bubble 3 was 5.7 mm/s, 7 mm/s and 4.5 mm/s respectively.

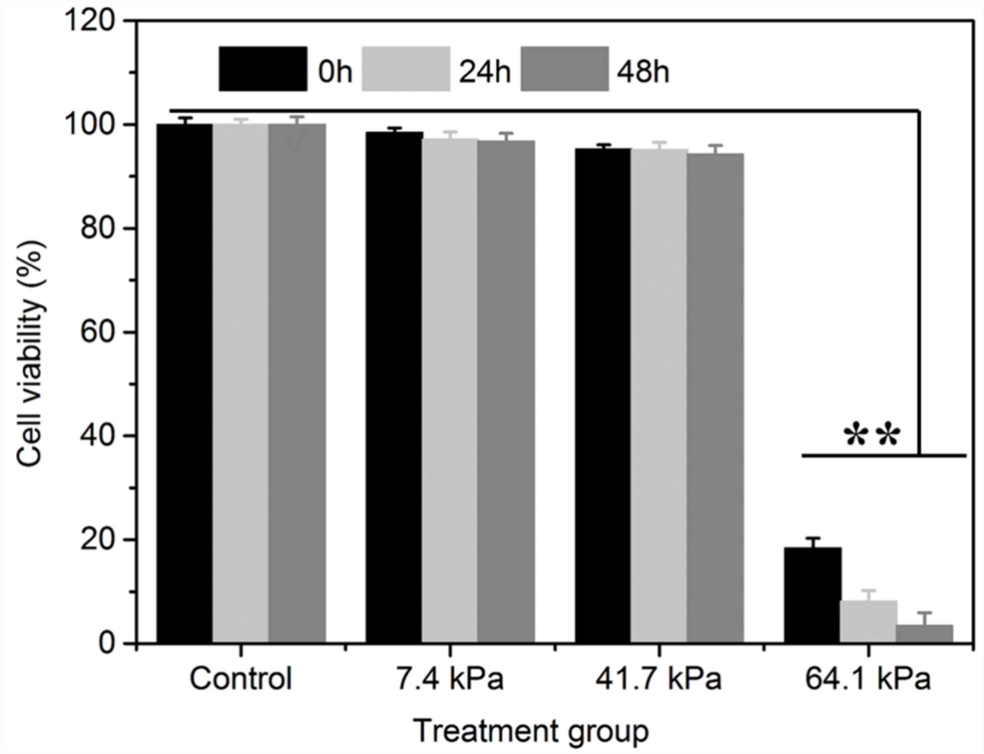

Figure S3 Treated cell viability analysis. To further investigate the long-term cell viability, the sonoporated cells are collected at the outlets and then cultured in 96 well plates. Cell counting Kit-8 (CCK-8, DOJINDO, Japan) is utilized to detect cell viability after 0 h, 24 h and 48 h. Compared to control group, the cell viability has no significant difference when the acoustic pressure was 7.4 kPa or 41.7 kPa. However, there is a significant difference in the group with acoustic pressure of 64.1 kPa. **$P<0.1$.

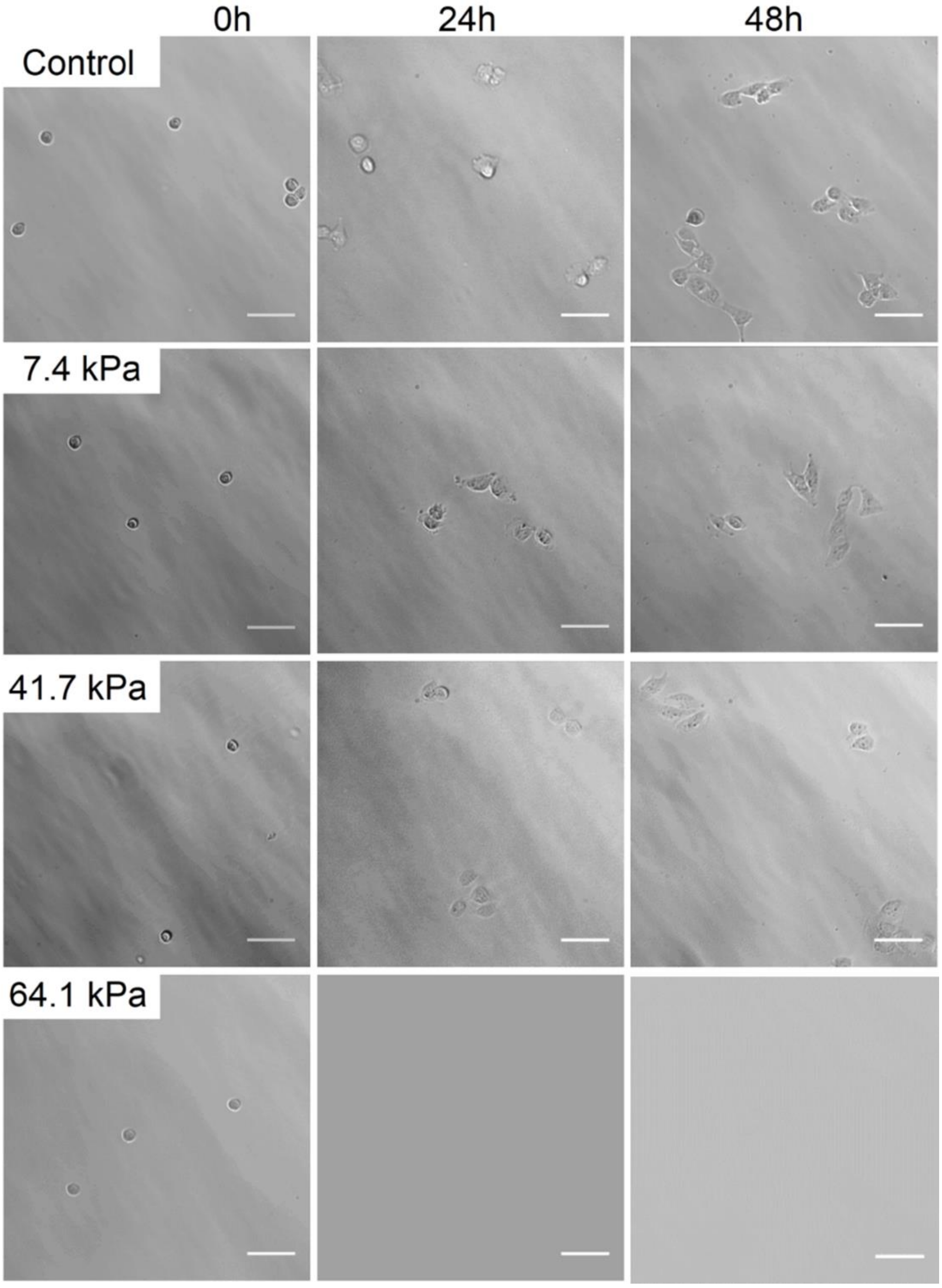

Figure S4 Treated cell morphology analysis. Besides the cell viability, the cell morphology is further investigated at the acoustic pressure of 7.4 kPa, 41.7 kPa and 64.1 kPa. For the acoustic pressure of 7.4 kPa, 41.7 kPa, the cells adhere to substrate and began to grow at 24 h. Cell proliferation is observed at 48 h. Instead, when the acoustic pressure is 64.1 kPa, almost all the cells are dead and no proliferation is observed at 24 h and 48 h.

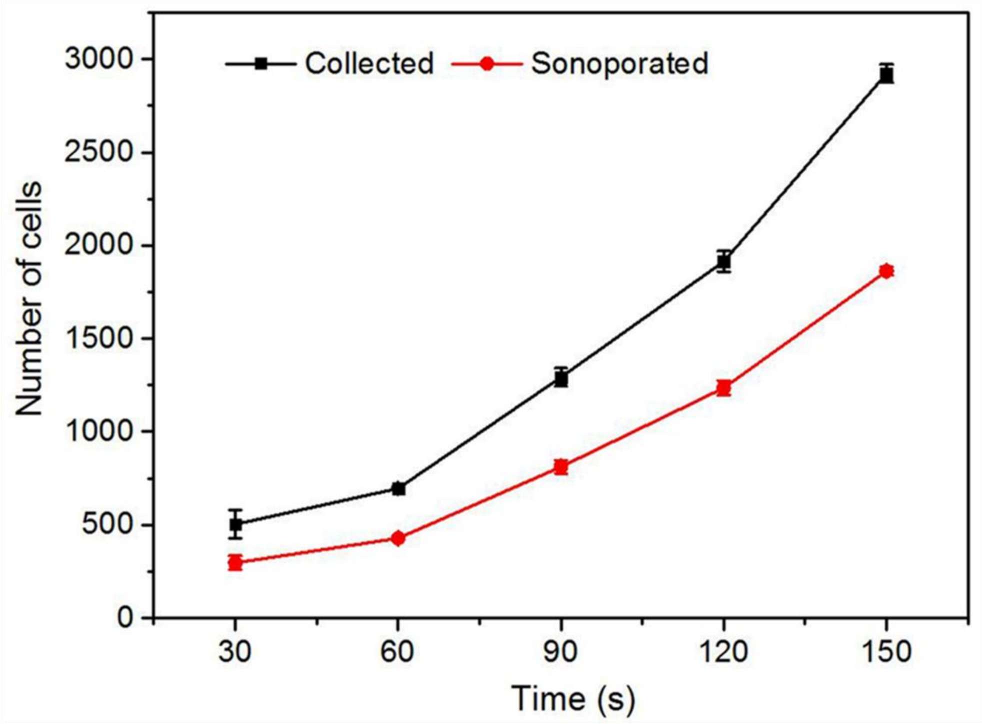

Figure S5 Throughput of the stable cavitation microbubble array. The result shows the sonoporated and collected cells over time when the acoustic pressure is held constant at 41.7 kPa. Within 60 s, the numbers of the sonoporated and collected cells are 429.6±3.6 and 696± 4.8, respectively. After 150 s, the sonoporated cells reach more than 1863.6. The potential efficiency of this parallel sonoporation platform can be further improved by bonding more microchannels on the glass substrate.

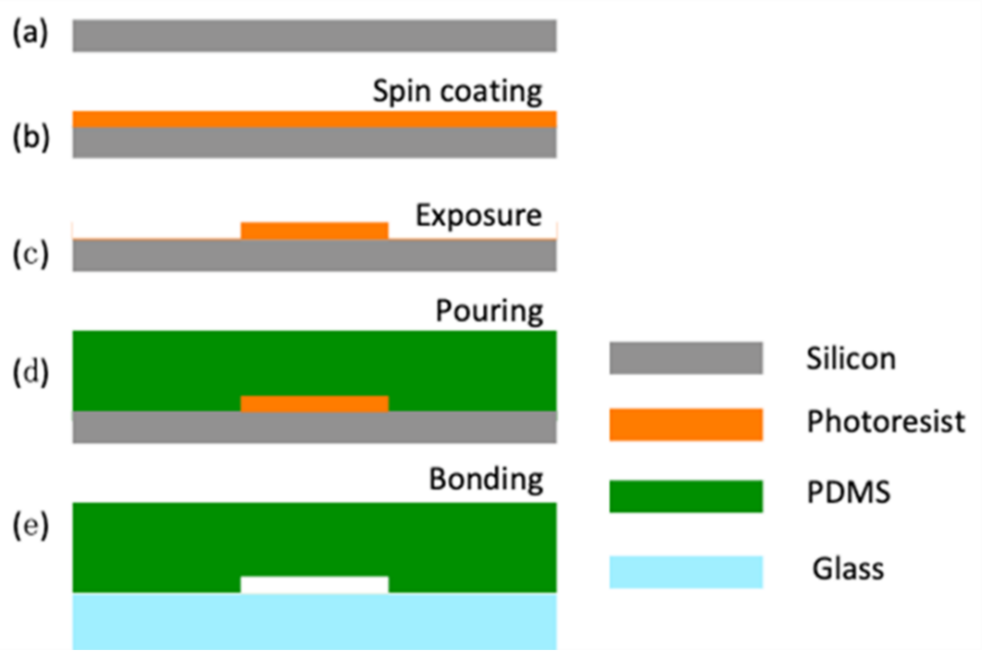

Figure S6 Fabrication process of stable cavitation microbubble array. (a-d) A soft-lithography process is used to fabricate multiple rectangular microcavities in the PDMS channel. (e) Bond the PDMS channel to the glass substrate by the plasma treatment.

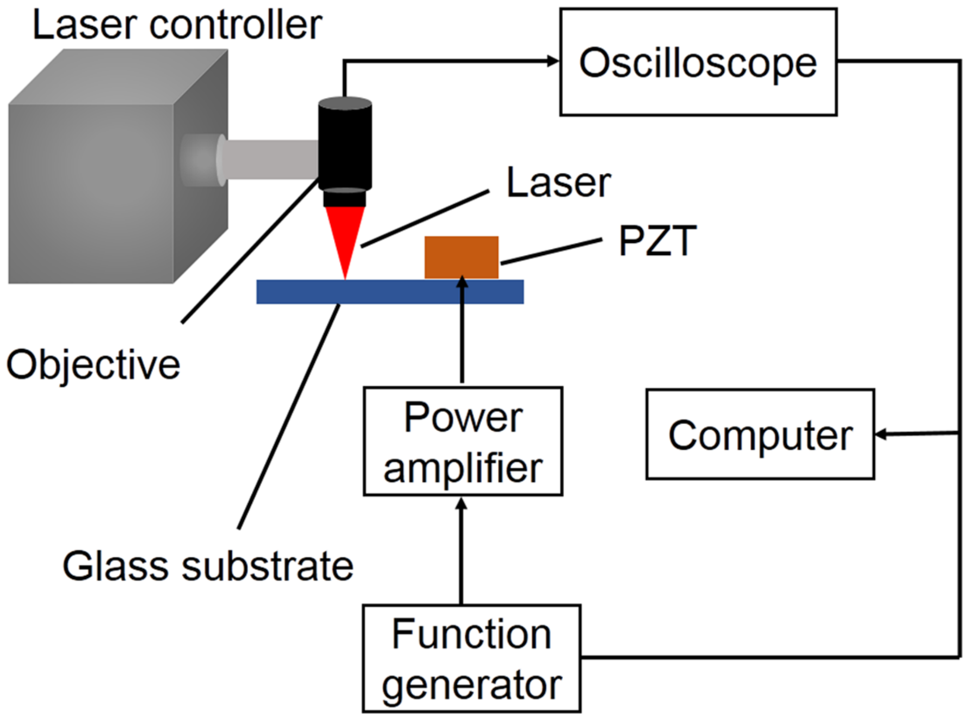

Figure S7 Schematic of the measurement vibration amplitude of the glass substrate by LDV system. The LDV is positioned perpendicular to the propagation direction of acoustic waves and is utilized to measure the vibration of substrate in a non-contact manner. The laser beam from the LDV is directed at the surface of the glass substrate, and the vibration amplitude is extracted from the Doppler shift of the reflected laser beam frequency due to the motion of the surface.

Table 1 The relationship among the input voltage applied to PZT, vibration amplitude of glass substrate and acoustic pressure.

Input voltage （V_{pp}）	Vibration amplitude (nm)	Acoustic pressure (kPa)
10	6.4	7.4
60	36.1	41.7
100	55.4	64.1
